# On the Influence Which Water Exerts on the Physical Properties of Various Solid Azotated (Animal) Substances

**Published:** 1821-11

**Authors:** 

**Affiliations:** Professor of Chemistry, &c.


### *On the Influence which Water exerts on the Physical Properties of various solid azotated (Animal) Substances.* By Mr. Chevreul, Professor of Chemistry, &c.

IN the course of my inquiries into the nature of animal matter, I have been led to examine a number of azotated substances, to which the presence of water imparts very remarkable properties. Some few of these substances have before attracted the notice of authors; but their number has never been increased; nor has any thing been said on the causes by which the presence of water in them seems regulated. Insulated facts alone have been recorded, in an inquiry which I think capable of supplying many interesting generalities in organic chemistry, and useful applications to animal physiology.

The first substances I studied under the particular points of view which form the object of the present memoir, are the tendons, the yellow elastic tissue of anatomists, fibrine, the cartilage of the external ear, the cartilaginous ligaments, the opaque cornea, and, lastly, albumine.

### Chapter I.—*On the Properties of the above-mentioned Substances in their dry and humid State; and on the Proportion of Water they lose when exposed to the Atmosphere, or placed into an exhausted Receiver previously dried by the Action of Sulphuric Acid.*

#### § I. *Of Tendons.*

The tendons diminish considerably in their volume by drying, particularly with respect to their thickness. They lose their whiteness, their satin gloss, their extreme suppleness; they acquire the semi-transparency of horn, and a reddish-yellow colour. They become stiff, lose much of their elasticity, and, when sufficiently twisted, they separate into small fibrous bundles, which are whitish in those parts where the air has penetrated among the fibres. That these properties, or physical characters, of the tendons in their recent state are due to the presence of a given quantity of water, which they lose on exposure to the atmosphere, is demonstrated by placing a dry tendon in distilled water. Under these circumstances, the tendon will absorb so much of that fluid after the lapse of twelve, or at most twenty-four hours, as to present again all the characters peculiar to fresh tendons; having, during this process, taken up as much, or nearly as much, water as by desiccation it had previously lost. This artificial *fresh* tendon may once more be dried, and again exposed to the action of water, when the same phenomena will take place. The experiment may be repeated a great number of times, without any loss of substance being perceived in the tendon. Some tendons of elephants, which have been kept in a dry state for four years, were transformed into *fresh* tendons by being kept immersed in water for a short time.

One hundred parts of a *thick* tendon of the elephant were reduced, by exposure to the air, to 51.56 parts; and, in the exhausted receiver, to 50 parts.

One hundred parts of a *thin* tendon of the same animal, under similar circumstances, were reduced in the first case to 46.91, and in the second to 43.36.

§ II. *Of the fresh Yellow Elastic Tissue.*

The fresh yellow elastic tissue is opaque and *neat*. Its structure is more decidedly fibrous than that of the tendon. Though formed of fibres, their disposition in the latter is such that, from their strong cohesion and the concentric layers of their arrangement, it is next to impossible to divide them into small bundles, like the fibres of the yellow tissue. With the exception of the colour, (which is rather of a deeper yellow,) and of its being semi-transparent and more fibrous, the yellow tissue, when desiccated, resembles the dry tendon. In this state, the yellow tissue has lost its property of extension when drawn out longitudinally, and of resuming afterwards its original dimensions: it has, therefore, no longer the essential character which distinguished it from the fresh tendon before desiccation. The proof that the elasticity of the fresh yellow tissue is due to water, is derived from its re-acquiring that property, after having lost it by desiccation, when immersed in water, of which it will absorb a quantity nearly similar to that it had parted with in drying.

One hundred parts of the yellow tissue of the elephant were reduced, by exposure to the air, to 52.57; and, in the exhausted receiver, to 50.5.

§ III. *Of the Cartilage of the External Ear.*

All the cartilages, when dry, consist of laminæ of unequal thickness, semi-transparent, of a reddish yellow colour, susceptible of being lacerated. When immersed in water, they absorb it, swell greatly, lose more or less of their colour and transparency, and become flexible.

One hundred parts of the cartilage of the external ear, after having been macerated in water, were reduced, by exposure to the air, to 33.35 parts; and, in a dry vacuum, to 30.64. The latter, being again put into water, absorbed 69.36 parts of that fluid, or nearly the same quantity they had lost by desiccation.

§ IV. *Of Cartilaginous Ligaments.*

One hundred parts of the cartilaginous ligament of the knee, after being macerated in water, were reduced to 26.41; and, in a dry vacuum, to 23.2. This last portion was again exposed to the action of water, and absorbed 74 parts of that fluid.

§ V. *Of Fibrine.*

This substance owes its white aspect, its flexibility, and slight elasticity, to the presence of water; for, when exposed to the air, it becomes semi-transparent, yellowish, inflexible, and loses all traces of elasticity! By the absorption of water, it regains all these properties. It is more difficult to ascertain the exact quantity of water present in fresh fibrine, than in any of the preceding substances, from the former being much more divisible in its texture.

§ VI. *Of the Opaque Cornea.*

The white milky aspect of this substance is due to the presence of water. It becomes transparent by desiccation, and resumes all its former properties by being made once more to absorb water.

§ VII. *Of the Albumine of Eggs.*

This substance has presented facts of the highest importance. Some of them have been noticed by preceding writers; but, for want of a proper value having been attached to them, none of those consequences have been deduced which would have naturally followed, and which are of great interest in animal chemistry.

Liquid albumine begins to coagulate, at the temperature of 61°, into a white opaline substance, the physical properties of which are generally known. The water, which held the albumine in solution, remains interposed between the parts which are become solid. If one hundred parts of liquid albumine be previously coagulated, and exposed to the air, they will be reduced to 15 parts: placed in a dry vacuum, they are further reduced to 13.65 of a colourless matter, slightly yellow, and semi-transparent.

Coagulated albumine, which has been dried, is susceptible, by the absorption of water, to regain all the physical properties of the white of egg which has been boiled; but, when reduced to 13.65 by desiccation, it only absorbs 68 parts, instead of 86.35, the quantity it had lost. This proves that the white of eggs boiled, holds an excess of water; and, in reality, it may be remarked, that the albumine which has been coagulated by heat in its shell, is invariably bedewed with moisture on its surface.

Having determined the proportion of water which coagulated albumine loses when exposed to the air, I felt anxious to know if albumine, which had not been previously coagulated, would lose the same quantity of water. I therefore took one hundred parts of albumine, taken from the same egg that had supplied me with the hundred parts of albumine used in the experiment after coagulation; and, after having exposed them to the air, I found that they had lost precisely the same quantity as before, —viz. 15 parts, and 13.8 when placed in a dry vacuum.

Under the circumstances last described, the albumine lost none of its transparency by the process of concentration; and, when dry, it had all the appearances of coagulated albumine that had been desiccated.

From the analogy of the physical properties, and, above all, from the result of the experiments with the coagulated and liquid albumine having been alike, I considered the two substances, when dried, to be identical; but, what was my surprise on finding that, when I added to the dry albumine that had not been previously coagulated, the 86.15 parts of water lost during concentration, it became wholly dissolved, and presented a liquid in every respect similar to the liquid white of the egg. Thomson, in his System of Chemistry, made nearly the same observation; but he does not seem to have drawn any useful inference from this curious fact.

It follows, from what has been stated respecting the weight of dry and coagulated albumine, that the commonly received explanation of the process of coagulation of that substance is unsatisfactory, and requires correction.

*Note.*—The above is only an abstract of a very interesting memoir of Mr. Chevreul, recently read at the Royal Institute of France. We thought it might prove satisfactory to a majority of our readers, to have the earliest intelligence respecting it; and we have therefore procured it from Paris, though it has not yet been published (to our knowledge,) in any of the scientific Journals. The second chapter, containing the applications of the above facts to animal physiology, will be given in our next Number.—A. B. G.

